# Sequential Therapy with Ropeginterferon Alfa-2b and Anti-Programmed Cell Death 1 Antibody for Inhibiting the Recurrence of Hepatitis B-Related Hepatocellular Carcinoma: From Animal Modeling to Phase I Clinical Results

**DOI:** 10.3390/ijms25010433

**Published:** 2023-12-28

**Authors:** Albert Qin, Chang-Ru Wu, Ming-Chih Ho, Chan-Yen Tsai, Pei-Jer Chen

**Affiliations:** 1 Medical Research & Clinical Operations, PharmaEssentia Corporation, Taipei 115, Taiwan; 2 Graduate Institute of Clinical Medicine, National Taiwan University College of Medicine, Taipei 100, Taiwan; 3 Department of Surgery, National Taiwan University Hospital, Taipei 100, Taiwan; 4 Hepatitis Research Center, National Taiwan University Hospital, Taipei 100, Taiwan

**Keywords:** hepatocellular carcinoma, HBV-related, anti-PD1 antibody, ropeginterferon alfa-2b, animal HBV model, clinical trial

## Abstract

Hepatocellular carcinoma (HCC) usually recurs after curative surgical resection. Currently, no approved adjuvant therapy has been shown to reduce HCC recurrence rates. In this study, the in vivo effect of sequential combination treatment with recombinant mouse interferon-alpha (rmIFN-α) and an anti-mouse-PD1 antibody on hepatitis B virus (HBV) clearance in mice was evaluated. A Phase I clinical trial was then conducted to assess the safety, tolerability, and inhibitory activity of sequential therapy with ropeginterferon alfa-2b and nivolumab in patients with HCC recurrence who underwent curative surgery for HBV-related HCC. The animal modeling study showed that HBV suppression was significantly greater with the rmIFN-α and anti-PD1 sequential combination treatment in comparison with sole treatment with rmIFN-α or anti-PD1. In the Phase I study, eleven patients completed the sequential therapy with ropeginterferon alfa-2b every two weeks for six doses at 450 µg, followed by three doses of nivolumab every two weeks up to 0.75 mg/kg. A notable decrease in or clearance of HBV surface antigen was observed in two patients. The dose-limiting toxicity of grade 3 alanine transaminase and aspartate aminotransferase increases was observed in one patient. The maximum tolerated dose was then determined. To date, no HCC recurrence has been observed. The treatment modality was well tolerated. These data support the further clinical development of sequential combination therapy as a post-surgery prophylactic measure against the recurrence of HBV-related HCC.

## 1. Introduction

Hepatocellular carcinoma (HCC) is a common and fatal cancer worldwide [1]. It is associated with underlying chronic liver pathological conditions, including chronic viral hepatitis. Chronic hepatitis B (CHB) contributes to more than 50% of global HCC cases [2,3]. Surgery is typically the curative treatment modality for early-stage HCC [4]. However, cancer recurrence is frequently observed. The tumor recurrence rate can be as high as 50–70% after five years [5,6,7]. Currently, there are no approved therapies for inhibiting the recurrence [7]. Therefore, effective and well-tolerated adjuvant therapies are urgently needed. 

Programmed cell death 1 (PD1) is a negative costimulatory receptor expressed primarily on the surface of activated T cells [8,9]. The binding of PD1 to its ligand, programmed cell death 1 ligand-1/2 (PD-L1/2), inhibits cytotoxic T cell-mediated immunological responses and elicits an immune checkpoint [10]. Tumor cells upregulate PD-L1 and utilize the PD1 pathway to evade T cell-mediated immune responses. Anti-PD1 and anti-PD-L1/2 therapeutic antibodies can interfere with the interactions between PD1 and its ligands, resulting in the enhancement of anti-tumor immunological response caused by cytotoxic T cells [10,11]. Anti-PD1 therapy causes a decline in or seroclearance of hepatitis B surface antigen (HBsAg) in patients with CHB [12]. HBsAg seroclearance is associated with a low risk of hepatitis B virus (HBV)-related HCC [13]. Anti-PD1 therapies have been approved for the treatment of advanced HCC, melanoma, metastatic non-small-cell lung cancer, and other advanced malignancies [14]. However, anti-PD1 therapies are also associated with known toxicities [15] and may not be suitable in post-surgical adjuvant settings for patients with HCC. A combination therapy that can potentially minimize toxicity and produce a significant anti-cancer effect may be applicable in this condition. 

Ropeginterferon alfa-2b represents a new-generation PEGylated interferon alpha (IFN-α)-based therapy with a favorable pharmacokinetic profile. It can be injected less frequently, for example, once every two weeks [16,17,18]. It has been approved for the treatment of polycythemia vera (PV), a myeloproliferative neoplasm (MPN), in the United States and Europe [19,20] and is currently under development for more approvals [21,22,23,24,25]. In its Phase II clinical trial, the application of 450 μg of ropeginterferon alfa-2b every two weeks demonstrated good tolerability and manifested anti-hepatitis B virus (HBV) effects [26]. In this study, we report our findings regarding an HBV mouse model sequentially treated with recombinant mouse-IFN-alpha (rmIFN-α) and an anti-mouse-PD1 antibody and a clinical Phase I study of sequential therapy with ropeginterferon alfa-2b and the anti-human PD1 antibody nivolumab in patients with HBV-related HCC after curative surgery. 

## 2. Results

### 2.1. Animal Modeling Data

**Figure 1:**
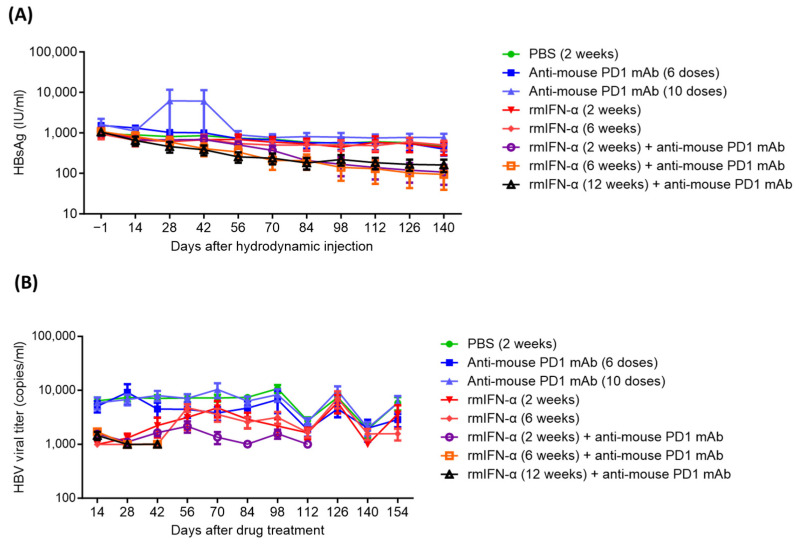
HBsAg (**A**) and HBV viral titer (**B**) in HBV-carrying CBA/CaJ mice.

An HBV mouse model (HBV-HDI) was generated via the intravenous injection of an HBV genotype A DNA plasmid into CBA/CaJ mice [27,28]. In this HBV-HDI mouse model, a sequential combination treatment with rmIFN-α and an anti-mouse PD1 antibody (RMP-17) continuously caused a decline in the mean HBsAg values compared with the phosphate-buffered saline (PBS) control. After sequential combination treatment, the mean HBsAg level was one log lower than that of the PBS control group (Figure 1A). In contrast, sole treatment with either rmIFN-α or anti-mouse PD1 showed no significant decline in the HBsAg level compared to the PBS control (Figure 1A). Compared with sole treatment with rmIFN-α or anti-mouse PD1, the sequential combination of rmIFN-α and anti-mouse PD1 antibody significantly reduced HBsAg levels and HBV viral titers in the HBV-carrying CBA/CaJ mice (Figure 1A,B). In addition, the HBsAg clearance rate was 44.4% (4/9) in mice receiving a 6-week treatment of rmIFN-α and anti-mouse PD1, which was higher than that observed in the groups receiving sole treatment with either rmIFN-α or anti-mouse PD1 (Table 1). The animal data showed a synergistic effect between rmIFN-α and anti-PD1 antibody for HBV suppression or even clearance in the HBV mouse model. No adverse effects on animal body weight, liver function, or blood cell production were observed.

### 2.2. Phase I Clinical Study

**Figure 2:**
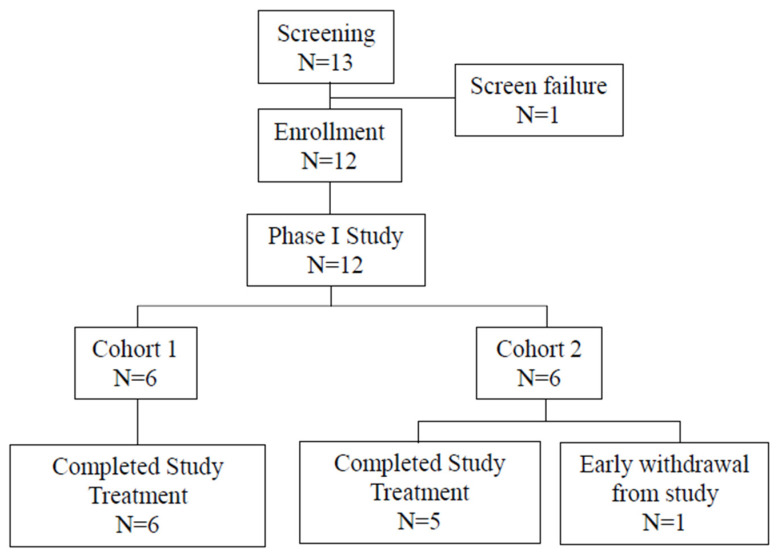
Summary of subject disposition in Phase I study.

A Phase I study was conducted to determine the maximum tolerated dose (MTD). A total of 12 eligible patients were enrolled in Cohorts 1 and 2 (Figure 2), with six patients in each cohort. Patients received six doses of ropeginterferon alfa-2b at 450 μg once every two weeks followed by three doses of nivolumab at 0.3 mg/kg in Cohort 1 and three doses of nivolumab at 0.75 mg/kg in Cohort 2. All patients completed the study treatment except for one who withdrew early because of grade 3 anorexia after receiving one dose of ropeginterferon alfa-2b in Cohort 2.

The mean age and standard deviation of the eligible patients were 61.8 and 10.3 years, respectively (Table 2). Six (50%) patients had liver cirrhosis before participating in the study. All patients experienced at least one adverse event (AE) after treatment. Most AEs were either mild or moderate (Table 3). No grade 4 or 5 AEs were observed. Similarly, no serious AEs (SAEs) were observed. Four patients (33.3%) experienced grade 3 AEs. The most frequent AE was pyrexia (50%), followed by alanine transaminase (ALT) increase (41.7%), aspartate aminotransferase (AST) increase (41.7%), fatigue (33.3%), and neutrophil count decrease (25%).

Dose-limiting toxicities (DLTs) were observed in one patient. Drug-related grade 3 ALT and AST increases were observed in one patient in Cohort 1. The patient completed the ropeginterferon alfa-2b treatment and received one dose of nivolumab. No DLTs were observed in Cohort 2. However, given that the DLTs of grade 3 ALT and AST increases were observed in Cohort 1 and that there were greater levels of grade 2 ALT and AST increases in Cohort 2, we determined that Cohort 2 reached the MTD of the study based on the overall safety assessment. Therefore, six doses of ropeginterferon alfa-2b at 450 ug followed by three doses of nivolumab at 0.75 mg/kg were determined to be the MTD for the adjuvant sequential combination therapy.

**Figure 3:**
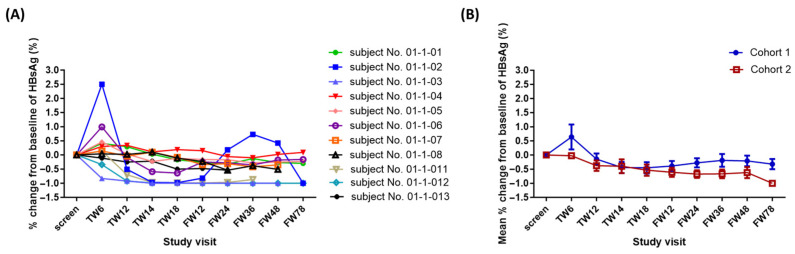
Percentage change in HBsAg in Phase I study. (**A**) Percentage change in HBsAg in each subject. (**B**) Mean percentage change in HBsAg.

To date, all patients are alive without cancer recurrence. The mean follow-up period was 716.75 days (minimum, 114 days; maximum, 1416 days). HBsAg was undetectable in one patient in Cohort 1 at follow-up week 12 and after (Figure 3A). The mean HBsAg levels decreased over time in Cohort 2 during the treatment period (Figure 3B).

## 3. Discussion

HCC is a common cause of cancer-related deaths, and most cases are associated with HBV infection. Patients with HCC are at a high risk of tumor recurrence after surgical resection. Currently, there are no approved therapies for inhibiting tumor recurrence. In this study, our animal modeling data demonstrated that sequential combination treatment with rmIFN-α and anti-PD1 antibody led to a synergistic effect in HBV suppression and clearance. Our Phase I clinical study further suggested that sequential combination therapy with ropeginterferon alfa-2b and nivolumab was well tolerated. This combination therapy can facilitate the clearance of residual HBV infection and inhibit cancer recurrence in patients with HBV-related HCC after curative surgery.

PD-L1 is often overexpressed during CHB infection [29,30,31]. In patients with HCC and CHB, nivolumab treatment decreases HBsAg levels [12,32]. HBsAg seroclearance has been previously suggested to be associated with a lower risk of late recurrence in HBV-related HCC [13]. Interrupting the PD1 signaling pathway can reverse T cell exhaustion, leading to the inhibition of viral infection and the proliferation of cancer cells [33,34,35]. Blockading PD1 signaling could also enhance IFN-gamma production and the proliferation of peripheral blood mononuclear cells and partially recover dysfunctional virus-specific B cells from CHB patients [36,37]. Nivolumab is approved for HCC treatment in combination with ipilimumab [38]. It is reasonable to assume that anti-PD1 antibodies suppress cancer recurrence in patients with HBV-related HCC who have undergone surgical resection. However, the toxicities associated with anti-PD1 treatment at approved dose levels and HBV reactivation due to the residual viral genome may pose hurdles for its sole use as a prophylactic measure against HCC occurrence [39,40,41].

Type 1 IFNs, including IFN-α and beta (IFN-β), share the same receptor components and induce similar biological activities [42,43,44]. They exhibit anti-proliferative, immunostimulatory, and anti-angiogenic activities [45,46,47,48]. The anti-proliferative effects include cell cycle inhibition and apoptosis [49,50,51]. In solid tumor cells, they can slow S-phase progression by activating an intra-S phase checkpoint and inducing senescence entry accompanied by a loss of tumorigenicity [52]. In addition, they stimulate the immune system to elicit anti-tumor activities, including the induction of natural killer cell-dependent and CD8+ T cell-mediated anti-tumor responses [53,54]. These combined anti-tumor activities can inhibit tumor formation. Pegylated IFN-α treatment has been shown to inhibit HBV and is approved for patients with CHB [55,56,57]. Its treatment is associated with a lower incidence of HCC among patients with HBV [58]. PEGylated IFN-α can also upregulate the chemokine CCL4 secreted by tumor cells and consequently recruit cytotoxic CD8+ T cells to infiltrate the HCC microenvironment, facilitating an improvement in the antitumor effect induced by anti-PD1 treatment [59]. In addition, IFN-α has been observed to reduce the glucose consumption of HCC cells and induce a high-glucose microenvironment that can foster the transcription of the T cell costimulatory molecule Cd27 in infiltrating CD8+ T cells and, consequently, potentiate the anti-PD1-induced immune response [60]. Therefore, a PEGylated IFN-α therapy with both anti-cancer and anti-HBV activities with the ability to enhance the anti-PD1-induced antitumor effect may reduce the need for anti-PD1 treatment at a high dose level when inhibiting HCC recurrence. Sequential combination treatment may potentially eradicate residual or newly formed tumor cells due to HBV infection in patients with HBV-related HCC after curative surgery. Our results suggest that sequential combination therapy with ropeginterferon alfa-2b and nivolumab may be a feasible and promising regimen for inhibiting cancer recurrence in patients with HBV-related HCC after curative surgery.

## 4. Materials and Methods

### 4.1. HBV-HDI Mouse Model

Six- to eight-week-old male CBA/CaJ mice were bred at the National Taiwan University Laboratory Animal Center. The mice were intravenously injected with 10 μg of HBV genotype A DNA plasmid dissolved in PBS equivalent to approximately 8% of the mouse’s body weight, as previously described [27,28]. Serum HBsAg and HBV DNA were measured to monitor HBV persistence. All the experiments were performed in accordance with the guidelines established by the Institutional Animal Care and Use Committee of the National Taiwan University College of Medicine.

### 4.2. Preclinical Materials

Anti-mouse PD1 (RMP-17) is a monoclonal antibody targeting mouse PD1 generated via hybridoma screening at the PharmaEssentia Corporation Research Laboratory. rmIFN-α was produced using the *Escherichia coli* expression system at PharmaEssentia Corporation.

### 4.3. Treatment of HBV-Carrying CBA/CaJ Mice

Mice were divided into eight groups to investigate the effect of rmIFN-α on sequential combination with anti-mouse PD1. The drug dosages and administration routes are described below.

Group 1 (control): Six HBV-carrying CBA/CaJ mice were subcutaneously (s.c.) injected with 200 μL of PBS every other day (Q2D) × 8 (days 0–14).

Group 2: Six HBV-carrying CBA/CaJ mice were s.c. injected with 800 IU/g of rmIFN-α (Q2D × 8, days 0–14).

Group 3: Six HBV-carrying CBA/CaJ mice were s.c. injected with 800 IU/g of rmIFN-α (Q2D × 22, days 0–42)

Group 4: Ten HBV-carrying CBA/CaJ mice were intraperitoneally (i.p.) injected with 32 μg/g of the anti-mouse PD1 antibody (Q2D × 6, days 16–26).

Group 5: Ten HBV-carrying CBA/CaJ mice were i.p. injected with 32 μg/g of anti-mouse PD1 antibody (Q2D × 10, days 16–34).

Group 6: Ten HBV-carrying CBA/CaJ mice were s.c. injected with 800 IU/g of rmIFN-α (Q2D × 8, days 0–14) and then i.p. injected with 32 μg/g of the anti-PD1 antibody (Q2D × 6, days 16–26).

Group 7: Ten HBV-carrying CBA/CaJ mice were s.c. injected with 800 IU/g of rmIFN-α (Q2D × 22, days 0–42) and then i.p. injected with 32 μg/g of the anti-PD1 antibody (Q2D × 6, days 44–54).

Group 8: Ten HBV-carrying CBA/CaJ mice were s.c. injected with 800 IU/g of rmIFN-α (Q2D × 43, days 0–84) and then i.p. injected with 32 μg/g of the anti-PD1 antibody (Q2D × 6, days 86–96).

### 4.4. Quantification of HBsAg and HBV DNA in Mice

Serum HBsAg and HBV DNA levels were quantified using the Abbott Architect I1000 system (Abbott Diagnostics, Green Oaks, IL, USA) and the Roche Lightcycler 480 (Roche Diagnostics, GmbH, Mannheim, Germany), respectively. The sequences of HBx-specific primers used to quantify the copy numbers of HBV DNA were 5′-CCGATCCATACTGCGGAAC-3′ (forward (nt 1261–1600)) and 5′-GCAGAGGTGAAGCGAAGTGCA-3′ (reverse) [61]. The detection limitation was 1000 copies/mL.

### 4.5. Clinical Materials:

Ropeginterferon alfa-2b was produced by the PharmaEssentia Corporation. It was provided as a prefilled syringe of 500 µg/1.0 mL. Nivolumab (OPDIVO^®^, Bristol-Myers Squibb Company, Brooklyn, NY, USA) was obtained via investigator prescription in a dosage form of 20 mg/2 mL or 100 mg/10 mL per vial.

### 4.6. Study Design

This clinical study was designed as a Phase I/II trial. The Phase I study aimed to evaluate the safety and tolerability and define the MTD of the sequential administration of ropeginterferon alfa-2b and nivolumab in patients who received curative surgery for hepatitis B-related HCC. The Phase II trial was designed to further evaluate the safety and prophylactic effect of the sequential administration of ropeginterferon alfa-2b and nivolumab at the MTD. Phase I was conducted at the National Taiwan University Hospital (NTUH), Taiwan (approval number: 201710061MIPB). The Phase I study was completed, but the Phase II study has not yet started.

Sequential administration of ropeginterferon alfa-2b and nivolumab was assessed using a 3 + 3 dose escalation scheme. Eligible patients were enrolled in four dose cohorts to receive six doses of ropeginterferon alfa-2b at a dose of 450 μg once every two weeks, followed by three doses of nivolumab every two weeks at a predetermined dose level based on the cohort, including 0.3 mg/kg for Cohort 1; 0.75 mg/kg for Cohort 2; 1.5 mg/kg for Cohort 3; and 3 mg/kg for Cohort 4. Ropeginterferon alfa-2b was administered via subcutaneous injection, and nivolumab was administrated through intravenous infusion over 60 min [62,63]. Patients were followed up with a site visit for an additional 48 weeks after completion of the study treatment. Disease progression was monitored using computed tomography (CT) or magnetic resonance imaging (MRI) at days 127, 211, 337, 463, and 673 after the first dose of P1101. Survival status was monitored continuously. All AEs were coded using preferred terms according to Medical Dictionary for Regulatory Activity (MedDRA) terminology. AE severity was graded using the National Cancer Institute (NCI) Common Terminology Criteria for Adverse Events (CTCAE), version 4.03.

### 4.7. Patients

Patients with HBV-related HCC who underwent surgical resection within eight weeks were enrolled. Other major inclusion criteria included positive results for HBsAg, undetectable HBV DNA, compensated liver disease, normal fundoscopic examination, and an Eastern Cooperative Oncology Group Performance Status score of 0 to 1. The major exclusion criteria included HCC that was not related to HBV; vascular invasion of HCC in imaging diagnosis; patients who had undergone transcatheter arterial embolization or chemoembolization, transcatheter arterial infusion, or chemolipiodolization in combination with surgery; and a concurrent active malignancy other than HCC.

## 5. Conclusions

Our animal data demonstrated a synergistic effect between rmIFN-α and anti-PD1 treatment for HBV suppression or even clearance. This effect was observed in patients with HCC who received sequential combination therapy with ropeginterferon alfa-2b and nivolumab in a Phase I clinical study. Most AEs were either mild or moderate. Increased liver transaminase was common but not associated with increased bilirubin levels or clinical symptoms. No unexpected AEs were observed. The MTD of sequential combination therapy with ropeginterferon alfa-2b and nivolumab was determined. Further exploration and clinical development of combination therapy are required.

## Figures and Tables

**Table 1:** Mean HBsAg and HBsAg clearance rate at the end of the animal study.

Treatment Groups	Mean HBsAg (IU/mL)	HBsAg Clearance Rate
PBS	471.9	0% (0/6)
Anti-PD1 (6 doses)	398.6	20% (2/10)
Anti-PD1 (10 doses)	769.1	10% (1/10)
rmIFN-α (2 weeks)	441.2	0% (0/6)
rmIFN-α (6 weeks)	504.7	0% (0/6)
rmIFN-α (2 weeks) + anti-PD1 (6 doses)	107.8 (*p*-value = 0.0012)	20% (2/10)
rmIFN-α (6 weeks) + anti-PD1 (6 doses)	94.3 (*p*-value = 0.0009)	44.4% (4/9)
rmIFN-α (12 weeks) + anti-PD1 (6 doses)	162.1 (*p*-value = 0.0040)	0% (0/10)

**Table 2:** Summary of demographics and baseline characteristics in the Phase I clinical study.

Characteristics	P1101 + Anti-PD1 N = 12		
	Cohort 1 n = 6	Cohort 2 n = 6	Total n = 12
Age, years			
Mean (SD)	64.2 (6.1)	59.5 (12.9)	61.8 (10.3)
Range (Min–Max)	53–72	40–75	40–75
Gender			
Male, n (%)	5 (83%)	4 (67%)	9 (75%)
Female, n (%)	1 (17%)	2 (33%)	3 (25%)
Liver Cirrhosis			
Yes	3 (50%)	3 (50%)	6 (50%)
No	3 (50%)	3 (50%)	6 (50%)

Max: maximal; Min: minimal; SD: standard deviation.

**Table 3:** Summary of adverse events (AEs) in the Phase I study.

AEs, n (%)	Cohort 1 (n = 6)			Cohort 2 (n = 6)			Total (n = 12)
Any AE	6 (100)			6 (100)			12 (100.0)
Any SAE	0 (0)			0 (0)			0 (0)
AEs occurring in >10% of patients n (%)	Grade 1	Grade 2	Grade 3	Grade 1	Grade 2	Grade 3	
Pyrexia	4 (66.7)	0 (0)	0 (0)	2 (33.3)	0 (0)	0 (0)	6 (50.0)
ALT increased	1 (16.7)	1 (16.7)	1 (16.7)	0 (0)	2 (33.3)	0 (0)	5 (41.7)
AST increased	1 (16.7)	1 (16.7)	1 (16.7)	0 (0)	2 (33.3)	0 (0)	5 (41.7)
Fatigue	2 (33.3)	0 (0)	0 (0)	2 (33.3)	0 (0)	0 (0)	4 (33.3)
Neutrophil count decreased	0 (0)	1 (16.7)	1 (16.7)	0 (0)	1 (16.7)	0 (0)	3 (25.0)
Decreased appetite	1 (16.7)	0 (0)	0 (0)	0 (0)	0 (0)	1 (16.7)	2 (16.7)
Insomnia	1 (16.7)	0	0 (0)	1 (16.7)	0 (0)	0 (0)	2 (16.7)

AE: adverse event; SAE: serious adverse event; ALT: alanine transaminase; AST: aspartate aminotransferase. Note: (1) % = percentage of patients with n as the denominator.

## Data Availability

Data will be available to external researchers upon reasonable request from the investigators and PharmaEssentia.
